# Hirsch Index and Truth Survival in Clinical Research

**DOI:** 10.1371/journal.pone.0012044

**Published:** 2010-08-06

**Authors:** Thierry Poynard, Dominique Thabut, Mona Munteanu, Vlad Ratziu, Yves Benhamou, Olivier Deckmyn

**Affiliations:** 1 University Pierre et Marie Curie Assistance Publique Hôpitaux de Paris Liver Center, Service d'Hépato-Gastroentérologie, Groupe Hospitalier Pitié-Salpêtrière, Paris, France; 2 Biopredictive, Paris, France; The University of Adelaide, Australia

## Abstract

**Background:**

Factors associated with the survival of truth of clinical conclusions in the medical literature are unknown. We hypothesized that publications with a first author having a higher Hirsch' index value (h-I), which quantifies and predicts an individual's scientific research output, should have a longer half-life.

**Methods and Results:**

474 original articles concerning cirrhosis or hepatitis published from 1945 to 1999 were selected. The survivals of the main conclusions were updated in 2009. The truth survival was assessed by time-dependent methods (Kaplan Meier method and Cox). A conclusion was considered to be true, obsolete or false when three or more observers out of the six stated it to be so. 284 out of 474 conclusions (60%) were still considered true, 90 (19%) were considered obsolete and 100 (21%) false. The median of the h-I was = 24 (range 1–85). Authors with true conclusions had significantly higher h-I (median = 28) than those with obsolete (h-I = 19; P = 0.002) or false conclusions (h-I = 19; P = 0.01). The factors associated (P<0.0001) with h-I were: scientific life (h-I = 33 for>30 years vs. 16 for<30 years), -methodological quality score (h-I = 36 for high vs. 20 for low scores), and -positive predictive value combining power, ratio of true to not-true relationships and bias (h-I = 33 for high vs. 20 for low values). In multivariate analysis, the risk ratio of h-I was 1.003 (95%CI, 0.994–1.011), and was not significant (P = 0.56). In a subgroup restricted to 111 articles with a negative conclusion, we observed a significant independent prognostic value of h-I (risk ratio = 1.033; 95%CI, 1.008–1.059; P = 0.009). Using an extrapolation of h-I at the time of article publication there was a significant and independent prognostic value of baseline h-I (risk ratio = 0.027; P = 0.0001).

**Conclusions:**

The present study failed to clearly demonstrate that the h-index of authors was a prognostic factor for truth survival. However the h-index was associated with true conclusions, methodological quality of trials and positive predictive values.

## Introduction

Science progresses via a series of paradigms that are held to be true until they are replaced by a better approximation of reality [1]. In surgery and medicine two studies have estimated that the half-life of truth for clinical conclusions in the literature is 45 years [2], [3]. We had tried to identify factors that were independently associated with this truth survival, and found only two, one expected (the negative conclusion of the publication) and one non-expected (the absence of meta-analysis in the methodology used) [3]. We therefore concluded that better prognostic factors should be found to better convince clinicians of the long term utility of evidence-based medicine [3], [4].

In the previous study, we did not analyze any author's related factor [3]. In the present study we hypothesized that publications with a first author having higher h-I which quantifies [5] and predicts an individual's scientific research output [6], [7], should have longer survival. An association between the h-I and truth survival could be also the proof of concept of using this type of method for validating such indexes. So far, the h-I has been validated using '“scientific achievement”, as defined by criteria which are finally very redundant: the number of citations [6], -peer review [8], -grant proposals [9] or quantitative performance measurements [10]-[11].

We used 474 previously assessed articles [3] with an identified first author, and in which the survival of the main conclusions were updated in 2009.

## Methods

### Summary of the initial study methodology [3]

#### Selection of articles

We identified original articles concerning cirrhosis or hepatitis in adults from 1945 to 1999 in 11 five year periods. The articles selection was stratified into 3 categories: *non-randomized studies, randomized trials and meta-analyses*. In each five year period we selected 20 non-randomized articles from two journals, 10 published in Lancet and 10 in Gastroenterology. In each period we tried to select 20 randomized trials on cirrhosis or hepatitis, 10 from Lancet and 10 from Gastroenterology. We chose these two journals because they have published clinical studies in hepatitis and cirrhosis since at least 1945, because they are peer- reviewed with a high level of selection and have a high impact factors greater than 10. A hand search was utilized to select articles from 1945 to 1985. As a true randomization was very difficult to organize we used a selection by order of publication inside each 5 year period. The first article of the period concerning cirrhosis or hepatitis was chosen, then the last of the period, then the second, and then the one before the last and so on up to 20 articles. From 1985 to 1999 we used PUBMED electronic search specifying the following “limits”: cirrhosis or hepatitis, human, Lancet or Gastroenterology. Abstracts were randomly downloaded using a similar selection method, stratified by five year periods. We selected the first abstract listed on the first electronic page, then the first on the last electronic page, then the last on the second electronic page, then the last on the page before the last and so on up to 20 articles.

In each period we tried to select 20 randomized trials on cirrhosis or hepatitis, 10 from Lancet and 10 from Gastroenterology. This was possible from 1970 to 1999. In the periods from 1945 to 1969 we selected all identified randomized trials whatever the journal, with a range from four (1945-1950) to 20 trials (1965-1969). From 1945 to 1982 we used the_ manual method the hand searching method as previously described [5]. From 1982 to 1985 we completed the random selection by hand searching and from 1985 to 1999 by PUBMED as described for non-randomized studies.For the meta-analyses, we used a hand searching method as described in the systematic review of meta-analyses [12]. To be included, meta-analysis should be based on trials in the field of hepatology and published as full papers before 2000. The following operational definition of meta-analysis was adopted: a study in which a computation of an overall treatment effect, based on the estimation of treatment effect in each trial, was performed, and reported with its 95% con- fidence interval or with the corresponding statistical test. Meta-analyses on childhood diseases were not included [12].

#### Selection of conclusion

The one conclusion from each abstract that seemed to best summarize the findings was copied to a database. Editing of these sentences was restricted to the rephrasing of outdated terminology and the elimination of redundant words.

#### Observers

Six hepatologists, called the observers, assessed the form which contained the selected conclusions in a random order. The observers were fulltime hepatologists from different subspecialties but working in a hospital and aged from 31 to 65 years. Observers were blind to the period, the journal, the authors, the method (meta-analysis, randomized, non randomized), and the methodological quality from which each conclusion was derived. They classified each conclusion into one of three categories: 1) still true in 2000 (updated in 2009), 2) obsolete but not false, 3) false.

#### Prognostic factors

The following seven factors were analyzed; 1) the design (meta-analysis, randomized trial, not randomized study); 2) the quality assessment of randomized trials and meta-analyses had been made independent of this study by one of us (TP) by means of scoring methods [13]-[15]; articles were rated as high quality when the score was greater or equal to the mean (12 for randomized trials, 27 for meta-analyses) and as low quality when lower than the mean. Non-randomized studies were classified as low methodological quality as there is no specific scoring method; 3) negative or positive conclusions; 4) the type of disease (hepatitis, portal hypertension, other); 5) the domain of clinical research (therapeutic, diagnostic or other study; other studies were defined as explanatory studies not assessing treatment or diagnostic tests); 6) the journal of publication (Lancet, Gastroenterology, other); and 7) the specialty (medicine or surgery).

#### Statistical analysis

A conclusion was considered to be true, obsolete or false when three or more observers out of the six stated it to be so. When there was a split decision 3 to 3 regarding conclusions being true-not true the final conclusion was considered to be true; these splits concerned 9 out of 474 (1.9%) articles. When there was a split decision 3 to 3 regarding conclusions being obsolete-non obsolete the final conclusion was obsolete; these splits concerned 26 articles out of 474 (5.5%). When the article was not classified as either true or obsolete it was considered as false. The half-life was calculated according to the Kaplan Meier method using the censored time as the duration between the year of publication to the year 2000 (updated in 2009). The censored time is the time at risk of being refuted or found to be obsolete. We analyzed the truth survival: if the conclusion was assessed to be still true the case was censored at the end of follow-up. If the conclusion was assessed to be false or obsolete it was considered as a failure. The comparison between factors used the two-sided logrank test and the multivariate analysis proportional hazard regression analysis.

### Hirsh index

The h-I of first authors was the main prognostic factor assessed in the present study. The h-I were assessed in the first 6 months of 2010. The h-I was originally computed using Google Scholar (“Google Scholar Universal Gadget”) for first authors. Because Google Scholar is not a perfect Gold Standard of estimating h-I, other methods were used. The commonness of last names can introduce a false estimate of the h-I [16] and therefore for the high risk names we used “liver” as a supplementary selection criteria in the Scholar research. As the Scholar research should be less performing for the oldest publications, the h-I was also assessed using the Scopus database for first authors of articles that were published after 1995,and using the ISI data-base. Only the authors still publishing after 1980 have been taken into account as the applicability of ISI search was very low in the older periods.

The date of the publication as well as the scientific age of the author (time between first and last publications) are mathematically associated with the h-I, which is cumulative, and increases over time [5], [8], [10], [16]-[17]. Therefore analyses were stratified according to the publication date (1945-1964, 1965-1979, 1980-1999), the rate of the h-I (h-I/scientific life in years) was estimated and the scientific life duration of the author was included in multivariate analyses.

The seven characteristics of studies [3] and two author characteristics associated with the h-I in the literature (gender of author, and place of residence) [10], [16]-[17] were analyzed as possible confounding factors in the prognostic analyses. The gender was unknown from the Scholar research and from the first name initials. We used the personal knowledge of coauthors and the details of first name given by Scopus.

### Updated methods

No change was made for the selection of articles, and methodological quality assessment. Observer conclusions were updated in 2009, that is with 10 years more of follow-up. One previous observer had retired, two had moved and two new ones agreed to participate (MM, DT). The observers were asked to modify their previous conclusions if necessary. A conclusion was changed when at least three observers out of the five stated it to be so. Five changes occurred, one previously false conclusion and one previously obsolete became true, two previously true became false and one became obsolete.

The main a priori endpoint was the prognostic value of the h-I (quantitative value) in the multivariate analysis including previously identified prognostic factors. The other “significant” P values were detailed when < = 0.10 and were described as NS if >0.10.

Statistical descriptions and analyses used non-parametric methods. Median was expressed with a 95% confidence interval. Multiple comparisons used the Kruskal Wallis variance analysis with Dunn s' multiple comparison test. In comparison with the previous analysis the same time-dependent analyses were used. [3] A modification was made for the estimated time of censoring for obsolete or false conclusion, according to a pertinent critique [18]. Very old publications that had been declared obsolete at the end of follow-up could cause the duration of survival to be overestimated if they were in fact been obsolete or false many years earlier. Therefore for each obsolete or false conclusion, we estimated the year in which it became obsolete or false. We added the duration of scientific life in the Cox proportional regression model as a covariate for adjusting the prognostic value of the h-I. The conclusions of the first analysis and the factors associated or not associated with truth survival did not change [19].

It was not possible to assess directly the h-I of the author at the time of publication (baseline h-I) for each article included in the present survey. However it was possible to estimate the baseline h-I using backwards the progression rate of the given h-I. For example a Scholar h-I = 81 in 2010 (h-I_2010_), for an author with a mean speed (h-speed) of 2.53, it was possible to extrapolate that for one article of the present database published in 1995 (h-I_baseline_) the h-I was at this baseline date: h-I_baseline_ = h-I_2010_- (h-speed × (2010-1995))  = 81-2.53x(15) = 43. This baseline h-I was also assessed in the prognostic analysis.

It has been suggested that for a special “outstanding category” of top-scientists, citation' indexes can reflect scientific “quality” [20]. Therefore we planned an analysis of “top-hepatologists” conclusions, using the cutoff which select the 30 highest h-I. Using h-Scholar the cutoff was h-I  = 60; this resulted in 33 articles (6.1%), as there was 4 ties at h-I  = 60. Using h-Scopus the h-I cutoff was 33 and for ISI 38.

We have not previously observed a prognostic value of studies according to criteria based on methodological quality scoring systems [3]. Recently Ioannidis proposed a classification of research findings in 9 classes of positive predictive values according to various combinations of power, ratio of true to not-true relationships and bias [4]. The details of this classification are available in Table S1. Therefore we planned an analysis using this classification in the multivariate prognostic analysis.

## Results

A total of 474 articles were included. The characteristics of included first authors are given in Table 1 and of the articles are given in Table 2, stratified by periods. There was a majority of articles published by residents of the US and UK before 1980, and by residents of continental Europe after 1980. A large majority of articles were published by male first authors, who were not surgeons, with a median scientific life of 30 years. The methodological quality, expressed according to scoring systems or predictive value, was much better since 1980.

**Table 1:** Hirsch index and characteristics of included first author.

Characteristics	Publication year			
	1945-1964	1965-1979	1980-1999	Total
Total selected	110	120	244	474
**Hirsch Index**				
**Scholar 2010**	13 (9-15)	23 (17-28)	30 (26-33)	24 (20-27)
Scholar baseline	0 (0-0)	1 (0-4)	6 (5-8)	3 (1-4)
Scopus*	NA	NA	16 (15-17)	NA
ISI**	8 (3-11)	11 (10-14)	21 (17-25)	17 (15-18)
Google Scholar/yr	0.76 (0.65-0.89)	0.70 (0.57-0.83)	1.08 (0.96-1.14)	0.87 (0.82-0.93)
Scopus/yr*	NA	NA	1.03 (1.00-1.14)	NA
ISI/yr**	0.19 (0.08-0.50)	0.32 (0.24-0.41)	0.72 (0.63-0.88)	0.58 (0.45-0.63)
**Residence**				
US	72	52	49	173
UK	20	22	31	73
Italy	1	3	49	53
France	0	5	31	36
Europe (other)	7	19	51	77
Asia	4	9	21	34
Other	6	10	12	28
**Gender**				
Male	106	111	212	429
Female	4	9	32	45
**Scientific life**				
First publication	1956 (1952-1957)	1968 (1967-1970)	1979 (1977-1980)	1970 (1967-1971)
Last publication	1976 (1968-1981)	2008 (2007-2008)	2009 (2009-2009)	2008 (2008-2008)
Scientific life	18 (11-27)	38 (34-38)	29 (28-31)	30 (28-32)

Quantitative data are expressed with median and 95% confidence interval.

NA =  Not Applicable.

*Scopus Hirsch index calculated only for article published after 1994.

**ISI Hirsch index applicable for 320 authors. The applicability was 31/110 (28%) for 1945-1964, 72/120 (60%) for 1965-1979 and 217/244 (89%) for 1980-1999.

**Table 2:** Characteristics of included original articles.

Characteristics	Publication year			
	1945-1964	1965-1979	1980-1999	Total
Total selected	110	120	244	474
***Authorship***				
**First author**				
Article with author publishing once	82	92	148	322
Articles with author publishing several studies	28	28	96	152
**First article**				
First article by author	94	105	181	380
Articles with repeated same author	16	15	63	94
***Article***				
**Journal**				
Lancet	43	40	77	160
Gastroenterology	41	49	89	179
Other	26	31	78	135
**Method**				
Non-randomized	80	60	80	220
Randomized trial	30	60	80	170
Meta-analysis	0	0	84	84
**Quality**				
**Score above median**				
yes	6	16	106	128
no	104	104	138	346
**Positive predictive value (Ioannidis)**				
≥20%	7	20	136	311
<20%	103	100	108	474
**Negative result**				
Yes	19	35	57	111
No	91	85	187	363
**Disease**				
Hepatitis	48	39	103	190
Portal hypertension	23	25	74	122
Other	39	56	67	162
**Subject**				
Treatment	56	66	172	294
Diagnosis	14	18	31	63
Other	40	36	41	117
**Specialty**				
Medicine	100	113	237	450
Surgery	10	7	7	24

In the year 2009, 284 out of 474 conclusions (60%) were still considered true, 90 were considered obsolete (19%) and 100 (21%) false. The half-life of truth was 45 years. The survival rate of conclusions was 85% (95%CI 83-89%) at 20 years and -52% (95%CI, 47-57%) at 40 years.

### The h-Index

The first author Scholar h-I (median; 95%CI) was 24 (20-27), with a range from 1 to 85, and an increase of 0.87 (0.82-0.93) h-I per year of scientific life. There was a skewed distribution, not normal, with 33 articles published by 21 authors with h-I values -of 60 or higher. For authors publishing after 1994, the h-I, estimated using Scopus, was 17 (15-20) with an increase of 1.13 (1.00-1.40) per year. For the period after 1980, the h-I estimated using ISI, was 21 (17-25) with an increase of 0.72 (0.63-0.88) per year. The median baseline h-I was 0 (0-0) before 1965 and 6 (5-8) after 1980.

### Factors associated with the h-I estimated using Google scholar

As expected the h-I was highly associated with duration of scientific life and recent publications (Table 3). Authors that had published after 1980 had a significantly higher h-I (30; 26-33); for those that had published earlier, the value was 23 (17-28) between 1965 and 1979, and 13 (9-15) between 1945 and 1964. There was no association between gender and the h-I.

**Table 3:** Hirsch index according to characteristics of included first author.

Hirsch Index						
	median (95% CI) or Spearman's rank coefficient; P value detailed if <0.05					
Characteristics	h-Scholar	Speed h-Scholar	h-Scopus 1995-2009	Speed h-Scopus	h-ISI 1980-2009	Speed h-ISI
	n = 474	n = 474	n = 227	n = 227	n = 217	n = 217
All	24 (20-27)	0.87 (0.82-0.93)	17 (15-20)	1.21 (1.07-1.50)	21 (17-25)	0.73 (0.63-0.88)
**Residence**						
US	28 (21-33)	0.94 (0.84-1.05)	17 (12-25)	1.31 (0.92-1.79)	25 (12-32)	0.97 (0.32-1.90)
UK	24 (16-31)	1.06 (0.80-1.36)	14 (3-22)	1.00 (0.33-1.64)	21 (12-28)	0.88 (0.57-1.16)
Italy	20 (20-29)	0.74 (0.45-1.03)	17 (15-21)	1.21 (1.07-1.50)	18 (17-26)	0.63 (0.39-0.63)
France	31 (20-40)	1.17 (0.83-1.6)	21 (16-31)	1.50 (1.14-2.21)	27 (18-46)	1.16 (0.89-1.51)
Europe (other)	20 (17-29)	0.70 (0.59-0.87)	11 (9-21)	0.79 (0.64-1.50)	19 (12-27)	0.63 (0.38-0.87)
Asia	24 (13-35)	1.08 (0.68-1.71)	20 (11-28)	1.43 (0.79-2.00)	29 (20-36)	1.11 (1.04-1.32)
Other	19 (17-24)	0.76 (0.54-0.89)	10 (3-15)	0.71 (0.21-1.07)	8 (0-16)	0.36 (0-0.73)
**Gender**						
Male	25 (21-28)	0.88 (0.83-1.00)	17 (15-20)	1.04 (0.57-1.15)	23 (18-26)	0.71 (0.63-0.87)
Female	19 (16-25)	0.79 (0.62-1.29)	15 (8-16)	1.21 (1.07-1.46)	15 (7-20)	0.84 (0.30-0.92)
**Scientific life**						
First publication date	0.01 (NS)	0.31 (<0.0001)	-0.04 (NS)	-0.04 (NS)	-0.10 (NS)	0.33 (<0.0001)
Last publication date	0.44 (<0.0001)	0.14 (0.003)	0.54 (<0.0001)	0.50 (<0.0001)	0.23 (0.0006)	0.20 (0.003)
Scientific life (year)	0.43 (<0.0001)	-0.27 (<0.0001)	0.26 (<0.0001)	0.25 (<0.0001)	0.13 (NS)	-0.35 (<0.0001)
**Authorship**						
First article by author	21 (18-25)	0.89 (0.81-1.00)	16 (13-19)	1.15 (1.00-1.36)	23 (18-25)	0.85 (0.70-0.92)
Articles with repeated same author	31 (25-38) (<0.0001)	0.85 (0.79-1.00)	15 (15-22)	1.07 (1.07-1.69)	18 (17-28)	0.63 (0.39-0.86)

Articles with true conclusions had significantly higher h-I (28; 24-31) than those with obsolete (19; 15-25; P = 0.002 vs. true) or false conclusions (19; 16-25; P = 0.01 vs. true) (Table 4). The same trends were observed for the h-I “rate” per year 0.97 (95%CI 0.84-1.07) for true conclusions, vs. 0.76 for obsolete (95%CI 0.62-0.86; P = 0.07 vs. true) and 0.90 (95%CI 0.71-1.07; NS vs. true) for false conclusions.

**Table 4:** Hirsch index according to characteristics of included original articles.

Hirsch Index						
	median (95% CI) or Spearman's rank coefficient; P value detailed if <0.05					
Characteristics	h-Scholar	Speed h-Scholar	h-Scopus 1995-2009	Speed h-Scopus	h-ISI 1980-2009	Speed h-ISI
**Journal**						
Lancet	22 (19-27)	0.83 (0.76-0.89)	16 (12-22)	1.21 (0.93-1.64)	21 (17-29)	0.79 (0.58-0.92)
Gastroenterology	27 (22-30)	1.00 (0.87-1.15)0.02	21 (17-23)	1.50 (1.29-1.64)	26 (20-30)	1.01 (0.74-1.13)
Other	20 (20-29)	0.84 (0.76-1.00)	15 (15-17)	1.07 (1.07-1.21)	17 (17-20)	0.63 (0.39-0.63)
**Method**						
Non-randomized	20 (17-24)	0.80 (0.71-0.94)	17 (15-21)	1.27 (1.07-1.50)	23 (18-27)	0.85 (0.64-1.09)
Randomized trial	30 (24-33) 0.0008	0.97 (0.87-1.07)	20 (13-25)	1.50 (1.00-1.86)	27 (17-31)	0.87 (0.60-1.00)
Meta-analysis	25 (20-38) 0.0003	0.85 (0.80-1.08)	15 (15-17)	1.07 (1.07-1.31)	17 (17-23)	0.63 (0.39-0.71)
**Quality**						
Yes	36 (31-41)<0.0001	1.08 (0.86-1.12) <0.0001	22 (16-22)*0.01	1.69 (1.21-1.71) 0.006	26 (20-31)	0.89 (0.63-0.93)
No	20 (19-23)	0.81 (0.71-0.85)	15 (14-17)	1.07 (1.00-1.25)	18 (17-22)	0.63 (0.43-0.81)
**Predictive value**						
≥20%	33 (25-38)<0.0001	1.08 (1.00-1.14) <0.0001	16 (15-22)	1.07 (1.00-1.57)	20 (17-25)	0.70 (0.63-0.89)
<20%	20 (19-24)	0.83 (0.76-0.94)	15 (12-19)	1.00 (0.80-1.27)	22 (17-26)	0.77 (0.58-0.90)
**Negative result**						
Yes	21 (18-29)	0.87 (0.81-1.00)	15 (12-17)	1.07 (0.92-1.31)	17 (13-23)	0.58 (0.41-0.85)
No	25 (20-28)	0.84 (0.74-1.00)	19 (16-21)	1.36 (1.14-1.57)	24 (18-27)	0.81 (0.63-0.91)
**Disease**						
Hepatitis	27 (20-29)	0.97 (0.84-1.08)	21 (17-25)	1.50 (1.25-1.79)	27 (21-30)	1.00 (0.82-1.13)
Portal hypertension	25 (20-31)	0.88 (0.75-1.08)	16 (15-21)	1.11 (1.07-1.50)	17 (17-25) 0.03	0.63 (0.39-0.63) 0.0003
Other	22 (18-25)	0.83 (0.76-0.94)	12 (11-17) 0.004	0.93 (0.79-1.31) 0.004	14 (12-23) 0.0005	0.51 (0.32-0.79)<0.0001
**Subject**						
Treatment	26 (20-31)	0.89 (0.84-1.00)	16 (15-21)	1.14 (1.07-1.50)	20 (17-25)	0.63 (0.62-0.87)
Diagnosis	26 (18-31)	0.97 (0.68-1.15)	17 (9-22)	1.21 (0.71-1.62)	26 (13-31)	0.83 (0.45-1.11)
Other	20 (14-25) 0.046	0.78 (0.68-0.97)	19 (15-22)	1.36 (1.15-1.64)	22 (16-27)	0.89 (0.53-1.13)
**Specialty**						
Medicine	24 (20-28)	0.86 (0.81-0.97)	17 (15-21)	1.21 (1.07-1.50)	21 (17-25)	0.78 (0.63-0.89)
Surgery	23 (10-53)	1.05 (0.45-1.12)	15 (6-22)	1.07 (0.46-1.69)	17 (8-31)	0.39 (0.20-8.00)
**Truth survival**						
True	28 (24-31) 0.002	0.97 (0.84-1.07) 0.01	17 (15-21)	1.21 (1.07-1.50)	22 (17-25)	0.71 (0.63-0.89)
Obsolete	19 (15-24)	0.76 (0.62-0.86)	15 (6-22)	1.08 (0.57-1.71)	18 (8-24)	0.63 (0.20-1.00)
False	19 (16-26)	0.90 (0.71-107)	19 (11-23)	1.36 (0.79-1.69)	26 (12-30)	0.88 (0.58-1.09)

**Figure 1:**
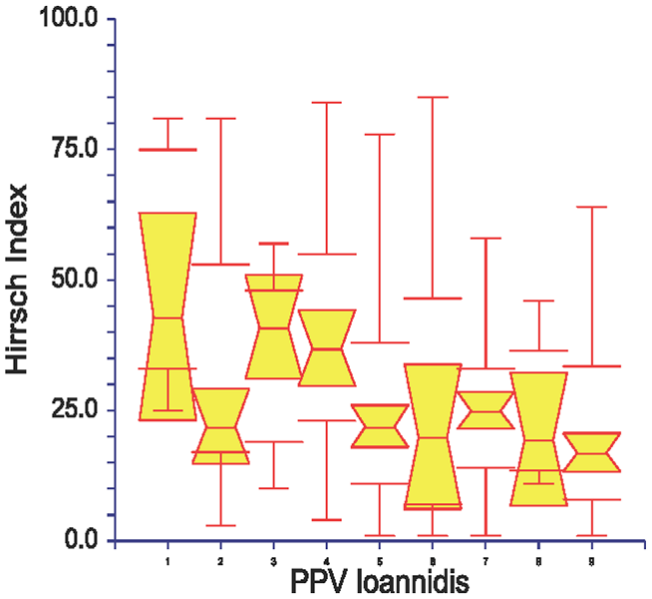
Hirsch-index and positive predictive value of research findings. On the vertical axis are plotted the Hirsch Index estimated using Google Scholar. On the horizontal axis are plotted the positive predictive value (PPV) of the conclusions of research findings, classified in 9 categories according to Ioannidis [4]: 1) Adequately powered randomized trial (RCT) with little bias and pre-study data (median h-I = 43); 2) Confirmatory meta-analysis of good quality RCTs (h-I = 22); 3) Meta-analysis of small inconclusive studies (h-I = 41); 4) Underpowered, but well-performed phase I/II RCT (h-I = 37); 5) Underpowered, poorly performed phase I/II RCT (h-I = 22); 6) Adequately powered exploratory epidemiological study (h-I = 20); 7) Underpowered exploratory epidemiological study (h-I = 25); 8) Discovery-oriented exploratory research with massive testing (h-I = 20); and 9) As 8, but with more limited bias (h-I = 17). Using Dunn's multiple comparisons test, the medians were significantly different between 1 and 5,7 and 9; 2 vs 9; 4 vs 5; 6 vs 7 and 9. Box plots were medians with 95% confidence intervals and extremes values.

Using univariate and not time-dependent analysis, the h-I was also associated with methodological quality either using scores (Table 4) or positive predictive value categories (Figure 1), randomization design, and with authors with several articles included (Table 5).

**Table 5:** Factors associated with truth survival.

	50 years truth survival percentage of studies without false or obsolete conclusion				
	Mean ± Standard error	Logrank	Significance (P)	Risk ratio (expβ)	Significance (P)
**h-Scholar 2010**					
>24 n = 232	50±5%	0.23	0.63	1.003 (0.994-1.011)	0.56
≤24 n = 240	46±4%				
**h-Scholar baseline**					
>3 n = 219	18±5%	29.8	<0.0001	1.027 (0.0131-0.041)	0.0001
≤3 n = 225	42±5%				
***h-Top-scientist***					
>60 n = 33	48±11%	6	0.10	0.57 (0.30-1.08)	0.09
≤60 n = 441	35±4%				
**h-Scopus** *					
>16 n = 116	53±8%	0.03	0.87	1.003 (0.984-1.022)	0.76
≤16 n = 111	48±9%				
**h-ISI** **					
>21 n = 107	52±9%	1.99	0.16	0.999 (0.990-1.010	0.98
≤21 n = 110	45±10%				
**Study conclusion**					
Negative n = 111	72±12%	20	<0.0001	0.41 (0.26-0.63)	0.0001
Positive n = 363	40±3%				
**Quality**					
**Methodological score**					
High n = 128	68±7%	0.1	0.71	1.08 (0.68-1.74)	0.71
Low n = 346	46±3%				
**Positive predictive value**					
≥20%	59±8%	0.2	0.65	0.87 (0.57-1.31)	0.50
<20%	34±4%				
**Disease**					
Hepatitis n = 190	50±5%				
Portal hypertension n = 122	53±6%	0.7	0.71	0.94 (0.65-1.33)	0.67
Other n = 103	41±5%				
**Type of research**					
Therapeutic n = 294	50±4%				
Diagnostic n = 63	18±7%	2.2	0.02	1.39 (0.95-1.99)	0.09
Other n = 117	40±6%				
**Journal**					
Gastroenterology n = 179	48±5%				
Lancet n = 160	43±5%	2.9	0.23	1.008 (0.75-1.36)	0.96
Other n = 135	53±6%				
**Specialty** ***					
Medicine n = 450	47±3%				
Surgery n = 24	54±11%	0.1	0.91	Not included	
**Methodology** ****					
Meta-analysis n = 84	87±2%				
Randomized trial n = 170	92±3%				
Non-randomized n = 220	87±3%	1.0	0.33	Not included	

*Analysis performed only for 244 articles published by authors still publishing after 1994.

**Analysis performed only for 217 articles published by authors still publishing after 1980 as there was too few authors with applicable h-ISI before 1980.

***Significance between h-ISI >17 and lower ***Not included in multivariate analysis as too small sample size for surgery articles.

****Survival analysis at 25 years because no meta-analysis was published before 1980. The squared correlation coefficient was 0.05 (P = 0.0004) for truth survival.

**Figure 2:**
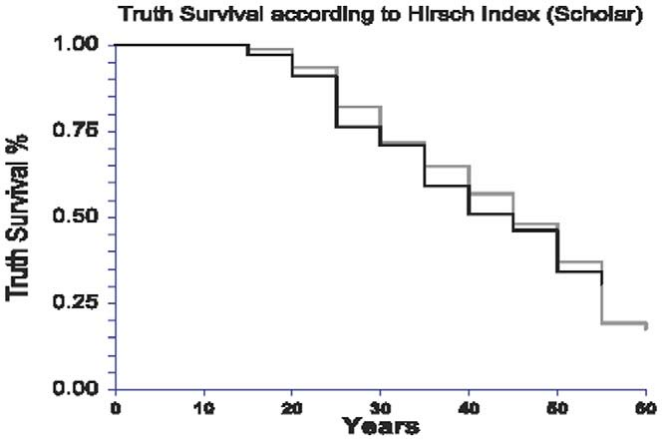
Survival of truth according to the Scholar Hirsch-index. Survival of 474 conclusions according to the h-index above the median value = 24, or not, using the Google Scholar data-base from 1945 to 2009. There was no significant difference between 50 years survival, 50±5% (black line) and 46±4% (grey line), respectively (P = 0.63).

**Figure 3:**
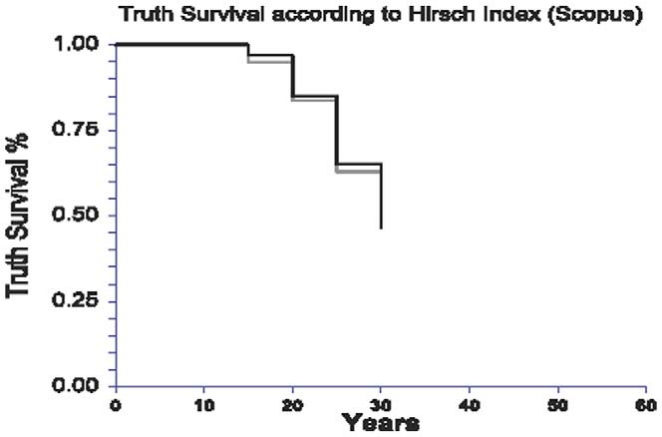
Survival of truth according to the Scopus Hirsch-index. Survival of 227 conclusions according to the h-index above of the median value = 16, using the Scopus data-base from 1995 to 2009. There was no significant difference between 50 years survival, 53±8% (black line) and 48±9% (grey line), respectively (P = 0.63).

There was no significant association between the h-I and truth survival using time-dependent analysis both in uni- and multivariate analyses (Table 5). Comparing the Scholar h-I there was no significant difference between 50 years survival (main end point), 50±5% (h-I above median) and 46±4% (under the median), respectively (P = 0.63) (Figure 2). There was also no difference in truth survival for Scopus h-I (Figure 3).

**Figure 4:**
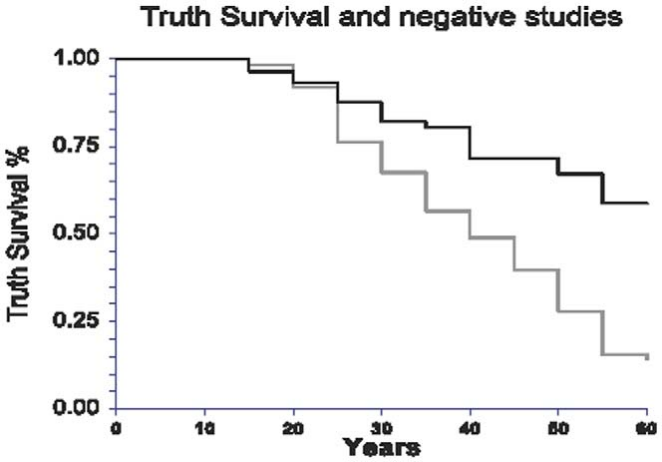
Survival of 474 conclusions according to the negative or positive finding. There was a significant difference between 50 years survival, 72±12% (black line) and 40±3% (grey line), respectively (P<0.0001).

For the main endpoint the risk ratio of the h-I was 1.003 (0.994-1.011) and was not significant (P = 0.56). There was a significant difference of the 50 years survival of conclusions according to the negative or positive finding, 72±12% (negative finding) and 40±3% (positive finding), respectively (P<0.0001) (Figure 4).

In a subgroup analysis restricted to 111 articles with negative conclusions we observed a significant independent predictive value of the h-I in multivariate analysis (risk ratio = 1.033; 95%CI, 1.008-1.059; P = 0.009). Negative conclusions of authors with an h-I >24 had an 82%±6% 50 years survival vs. 65%±9% for those < = 24 (NS). The observed difference was even greater among the Lancet's studies: 74%±16% vs. 47%±18% (NS).

The 50 year survival of the 30 higher h-I “outstanding category” conclusions was 48% (95% CI 29-73%) vs. 35% (28-42%, P = 0.10) among the others. Using Scholar h-I and ISI h-I the 25 year survival of the 30 higher vs others were 67% (44-90%) vs 60% (50-71%; P = 0.89) and 71% (51-90%) vs 59% (49-70%; P = 0.73).

### Concordance between the h-index estimated using Google Scholar, Scopus, and ISI

Concordance between the h-I estimated using Google scholar on the overall scientific life of authors and the h-I estimated using Scopus and ISI for the scientific life after 1994, was assessed for the 217 authors of articles published after 1994 and applicable ISI (1à not applicable out of 227). There was a highly significant concordance between the 3 h-I estimates. The Spearman's rank correlation between Scholar and Scopus was 0.72, between Scholar and ISI 0.81 (P<0.0001) and between Scopus and ISI 0.82 (all P<0.0001). The median h-I Scholar value was 31 (95%CI, 28-36) with a median of 29 years (95%CI 28-32) of scientific life; the median h-I Scopus value was 17 (95%CI, 15-21) with a median of 15 years (95%CI, 15-15) of scientific life; the median h-I ISI value was 21 (95%CI, 17-25). The rate of h-I per year was 1.08 (95%CI, 0.96-1.13) according to Scholar 1.13 (95%CI, 1.00-1.40) according to Scopus and 0.73 (95%CI 0.63-0.88). The classification of authors that ranked above/under the h-I median by Scholar (>31), by Scopus (>17) or by ISI (>21) had a high kappa concordance rate of  =  Scholar/Scopus 0.61 (SE = 0.07; P<0.001), Scholar/ISI 0.69 (SE = 0.07; P<0.001) and Scopus/ISI 0.85 (SE = 0.07; P<0.001). For h-I rate above/below 1 per year, the kappa were Scholar/Scopus 0.45 (SE = 0.06; P<0.001), Scholar/ISI 0.59 (SE = 0.06; P<0.001) and Scopus/ISI 0.31 (SE = 0.06; P = 0.01). In comparison with h-I estimated using Google Scholar, the h-I estimated using Scopus or ISI had similar variability according to characteristics of included first author (Table 3) and original articles (Table 4), and were also not independently associated with truth survival (Table 5).

### Predictive value of baseline Scholar h-index

For baseline H-I the prognostic value was opposite between uni and multivariate analyses. Using univariate comparison (Table 5), article with author baseline h-I greater than 3 (the median value) had lower 50 year survival (18%) than article with lower baseline h-I (42%; P<0.0001) and in multivariate analysis the quantitative value was positively associated with survival (Risk ratio = 0.027; P = 0.0001). This discrepancy was due to a very significant period effect. After 1980 the 25 year survival of author with baseline h-I >3 was 66% (54-77%) versus 63% (50-76%; NS) in h-I≤3, with in multivariate analysis a significant positive prognostic value (risk ratio = 0.027; P = 0.0001). Before 1980 the 25 year survival of author with baseline h-I >3 was 19% (5-34%) versus 63% (50-76%; NS) in h-I≤3 (negative prognostic value), with in multivariate analysis a significant positive prognostic value (risk ratio = 1.052; P = 0.04).

## Discussion

We observed that the h-I at the end of the study was associated with true conclusions, but its prognostic value did not survive with time-dependent analysis as previously observed for methodological quality. On the contrary baseline h-I (when the paper was written), was significantly and independently associated with truth survival, when adjusted on other covariables. Negative conclusions remained a robust and independent predictor of truth survival [3].

### Strength

We confirmed in the present study the intriguing prognostic value of negative conclusions (72% vs. 40% for 50 years survival for positive conclusions), which persisted after other factors had been taken into account. This prognostic value was not due to obsolete conclusions as among negative conclusions, as only 2% of negative conclusions had been rated as obsolete compared to 25% of positive conclusions. We found few negative studies which had been published in order to reveal previous false positive conclusions (Proteus phenomenon) [21]. An example is the article which concluded that hepatitis B virus was not responsible for primary biliary cirrhosis which was published 18 months after another article had suggested this association [3]. There was no significant difference in the h-I of authors with negative (h-I = 21) or positive (h-I = 25) conclusions. If we accept that most published research findings are false [4], the better survival of negative findings (“no relationships”) is a corollary of this statement. This is therefore the most plausible explanation of the better long term survival of negative findings.

Subgroup analyses are hazardous, but in a multivariate analysis restricted to 111 articles with negative conclusions we observed a significant independent predictive value of the h-I. This retrospective observation without a priori hypothesis must be confirmed by another study. We previously observed in the present cohort that the prognostic value of negative versus positive conclusions was mainly due to high differences among the randomized trials' conclusions: 68±13% for 52 negative conclusions compared with 14±4% (P<0.001) for 118 positive conclusions [3]. One hypothesis is that authors with an elevated h-I are principal investigators of “better trials” with better findings survival than those of authors with a lower h-I. From our analysis we cannot conclude that this “author effect” is a cause or a consequence of scientific performance. Some authors may be supported more by industry for other reasons than their “intrinsic” quality. A means of verifying whether “an intrinsic” author exists would have been to assess the factors associated with survival among articles published at the beginning of the authors' scientific life.

### Limitations

Our study has significant limitations. The study is retrospective between 1945 and 2000 and only prospective for the last 10 years of follow-up (updated in 2009). The inclusion criteria selected authors who may not have been representative of the overall biomedical community. They had published articles on liver diseases with high methodological levels (majority of randomized trials) in two competitive journals (mainly Lancet and Gastroenterology) with high impact factors in 2008, 28.4 and 12.6, respectively. We also used methods to assess methodological quality which are not the most recent and valid ones.

This selection should explain the high observed h-I (median of 24 for all periods and 30 for the period of 1980-1999). The h-I cannot be compared between different scientific fields or between different periods of publications [16], [17]. However, the observed median (h-I = 30) is higher compared with h-I of the same medical fields: versus other medical faculty members (same period): 7.6 mean h-I in 826 US oncologists [22], median 10 for 29 Dutch professors in cardiology [23], and median 23 for 45 editorial board members [24]. Because of this rather high h-I level, it is possible that our study suffered lack of power to demonstrate a prognostic significance of the h-I in multivariate analysis. “Top scientists” -according to the h-I were at the borderline of the prognostic value (Table 3). Enlarging the spectrum of authors could test this risk of error.

There is no gold standard for scientific truth definition. We used a definition that was decided by the majority vote of a panel of 5 experts, 10 to 65 years after the findings' publication. The main advantage was the duration of follow-up with subsequent progresses in the field of knowledge. The main weakness was the arbitrary choice of experts. To limit the risk of bias, the experts were chosen from different domains of Hepatology and had different ages [3]. We also adjusted the prognosis analysis using the classification of studies according to positive predictive values per Ioannidis [4]. The results were similar to the previous adjustments using the -validated quality scoring system of randomized trials and meta-analyses [3]. However we think that the positive predictive value estimates could be improved for negative findings and for diagnostic studies, which is a growing part of clinical research.

The h-I estimates had limitations and we cannot rule out that these limitations might be able to explain the absence of clear and independent prognostic values [7]–[10], [16]–[17], [ 25]. The first limitation is the reliability of a citation index in oldest years (1945-1980) before the prospective existence of PubMed and Google Scholar. The second main limitation is the commonness of last names which could introduce false estimates of the h-I. However, with the high risk names we used “liver” as a supplementary selection criterion in Scholar research and checked the authorship twice using Scopus for authors still publishing after 1994. Moreover, the main results were similar using two other estimates, Scopus and ISI (Table S2), which were significantly concordant.

Finally the extrapolation of baseline h-I at the year when the paper was written suggest a clear and independent prognostic value of h-I. The main limitation of this index in comparison with the 2010 h-I estimates, is its indirect assessment. This extrapolation rely on the normality and linearity of the h-I progression rate. We used median to reduce the risk of variability but a real prospective validation of the h-I prognostic value is needed.

### Conclusion

The h-I is simple, probably more accurate than other citation indexes for estimating authors' scientific outputs, and it is accepted when its limitations are understood [25], with [26] or without [7], [10] irony. We agree with Horne et al, that retaining a dignified aloofness to the h-I could be difficult for those with scores of less than 30 [7].

For living hepatologists, at least, our conclusions were balanced. The present study failed to clearly demonstrate that the h-index of authors was a prognostic factor for truth survival. However the h-I was partly validated as associated with true conclusions, the methodological quality of trials and with positive predictive values combining power, ratio of true to not-true relationships and bias.

Furthermore an indirect (extrapolated) estimate of baseline h-I clearly observed a high and independent prognostic value for articles published after 1980. Prospective study in the next decades should be initiated to confirm this observation.

## Supporting Information

Table S1 (0.07 MB DOCX)

Table S2 (0.09 MB DOCX)
