# Supplementary material for: Hirsch Index and Truth Survival in Clinical Research
Source: PLoS One. 2010 Aug 6;5(8):e12044. doi: 10.1371/journal.pone.0012044 (PMC2917363; doi:10.1371/journal.pone.0012044)
Supplement: Table S1 — (0.07 MB DOCX) [file pone.0012044.s001.docx]

Supplementary Table

| Positive Predictive Values (PPV) of Research Findings for Various Combinations of Power (1 − β), Ratio of True to Not-True Relationships (R), and Bias (u). Adapted from Ioannidis JPA, PlosMedicine 2005. | | | | |
| --- | --- | --- | --- | --- |
|  | | | | |
| **1− β** | **R** | **u** | **Practical Example** | **Positive Predictive Value** |
|  | | | | |
| 0.80 | 1:1 | 0.10 | Adequately powered RCT with little bias and 1:1 pre-study odds | 0.85 |
| 0.95 | 2:1 | 0.30 | Confirmatory meta-analysis of good quality RCTs | 0.85 |
| 0.80 | 1:3 | 0.40 | Meta-analysis of small inconclusive studies | 0.41 |
| 0.20 | 1:5 | 0.20 | Underpowered, but well-performed phase I/II RCT | 0.23 |
| 0.20 | 1:5 | 0.80 | Underpowered, poorly performed phase I/II RCT | 0.17 |
| 0.80 | 1:10 | 0.30 | Adequately powered exploratory epidemiological study | 0.20 |
| 0.20 | 1:10 | 0.30 | Underpowered exploratory epidemiological study | 0.12 |
| 0.20 | 1:1,000 | 0.80 | Discovery-oriented exploratory research with massive testing | 0.0010 |
| 0.20 | 1:1,000 | 0.20 | As in previous example, but with more limited bias (more standardized) | 0.0015 |

The estimated PPVs (positive predictive values) are derived assuming α = 0.05 for a single study. RCT, randomized controlled trial.
