# Supplementary material for: Hirsch Index and Truth Survival in Clinical Research
Source: PLoS One. 2010 Aug 6;5(8):e12044. doi: 10.1371/journal.pone.0012044 (PMC2917363; doi:10.1371/journal.pone.0012044)
Supplement: Table S2 — (0.09 MB DOCX) [file pone.0012044.s002.docx]

**Table S2: All h-Scopus according to characteristics of first author and original articles:**

|  | **Hirsch Index** | |
| --- | --- | --- |
|  |  | |
| **Characteristics** | **h-Scopus** | **Speed h-Scopus** |
|  | n=244 | n=244 |
|  |  |  |
| **Residence** |  |  |
| US | 17 (12-25) | 1.31 (0.92-1.79) |
| UK | 14 (3-22) | 1.00 (0.33-1.64) |
| Italy | 17 (15-21) | 1.21 (1.07-1.50) |
| France | 21 (16-31) | 1.50 (1.14-2.21) |
| Europe (other) | 11 (9-21) | 0.79 (0.64-1.50) |
| Asia | 20 (11-28) | 1.43 (0.79-2.00) |
| Other | 10 (3-15) | 0.71 (0.21-1.07) |
| **Gender** |  |  |
| Male | 17 (15-20) | 1.04 (0.57-1.15) |
| Female | 15 (8-16) | 1.21 (1.07-1.46) |
| **Scientific life** |  |  |
| First publication date | -0.04 (0.53) | -0.04 (0.53) |
| Last publication date | 0.54 (<0.0001) | 0.50 (<0.0001) |
| Scientific life (year) | 0.26 (<0.0001) | 0.25 (<0.0001) |
| ***Authorship*** |  |  |
| **First article** |  |  |
| First article by author | 16 (13-19) | 1.15 (1.00-1.36) |
| Articles with repeated same author | 15 (15-22) | 1.07 (1.07-1.69) |
| ***Article*** |  |  |
| **Journal** |  |  |
| Lancet 160 | 14 (11-20) | 1.08 (0.79-1.5) |
| Gastroenterology 179 | 20 (15-22) | 1.43 (1.07-1.57) |
| Other 135 | 15 (15-16) | 1.07 (1.07-1.14) |
| **Method** |  |  |
| Non-randomized | 17 (13-21) | 1.21 (0.93-1.50) |
| Randomized trial | 18 (11-24) | 1.33 (0.79-1.71) |
| Meta-analysis | 15 (15-17) | 1.07 (1.07-1.21) |
| **Quality** |  |  |
| Yes | 22 (16-22)*0.007 | 1.57 (1.14-1.69)0.003 |
| No | 15 (12-15) | 1.07 (0.92-1.15) |
| **Negative result** |  |  |
| Yes | 15 (11-15) | 1.07 (0.79-1.07) |
| No | 17 (15-21) | 1.21 (1.07-1.50) |
| **Disease** |  |  |
| Hepatitis | 19 (15-24) | 1.43 (1.21-1.79) |
| Portal hypertension | 15 (15-19) | 1.07 (1.07-1.37) |
| Other | 12 (10-15) 0.01 | 0.92 (0.71-1.21) 0.01 |
| **Subject** |  |  |
| Treatment | 15 (15-18) | 1.07 (1.07-1.36) |
| Diagnosis | 16 (9-22) | 1.14 (0.69-1.62) |
| Cognitive | 17 (12-21) | 1.25 (0.86-1.50) |
| **Specialty** |  |  |
| Medicine | 15 (0-22) | 1.14 (1.07-1.36) |
| Surgery | 16 (15-18) | 1.07 (0.00-1.69) |
| **Truth survival** |  |  |
| True | 16 (15-19) | 1.15 (1.07-1.43) |
| Obsolete | 14 (6-22) | 1.04 (0.46-1.69) |
| False | 15 (8-22) | 1.07 (0.57-1.64) |
